# Wide-field choroidal thickness in myopes and emmetropes

**DOI:** 10.1038/s41598-019-39653-w

**Published:** 2019-03-05

**Authors:** Hosein Hoseini-Yazdi, Stephen J. Vincent, Michael J. Collins, Scott A. Read, David Alonso-Caneiro

**Affiliations:** Contact Lens and Visual Optics Laboratory, School of Optometry and Vision Science, Queensland University of Technology, Brisbane, Australia

## Abstract

There is a paucity of knowledge regarding the normal *in-vivo* thickness of the choroid beyond the macula (~17°). In this study, the choroidal thickness of 27 healthy young adults was examined across the macular (the central 5 mm including the fovea, parafovea, and perifovea) and extra-macular (a 5–14 mm annulus including the near-periphery and periphery) regions using wide-field optical coherence tomography, and compared between emmetropes (n = 14) and myopes (n = 13). The choroid progressively thinned beyond the parafovea (350 ± 86 µm) towards the periphery (264 ± 44 µm), and was thickest superiorly (355 ± 76 µm) and thinnest nasally (290 ± 79 µm). Choroidal thickness also varied with refractive error; myopes exhibited a thinner choroid than emmetropes in the macular region (311 ± 88 vs. 383 ± 66 µm), however, this difference diminished towards the periphery (251 ± 48 vs. 277 ± 37 µm). Meridional variations in choroidal thickness were not different between myopes and emmetropes. In conclusion, the choroid was thickest within the perifovea; thinned substantially towards the periphery, and exhibited the minimum and maximum peripheral thinning superiorly and nasally across a 55° region respectively. Choroidal thinning associated with myopia was more pronounced in the macular than extra-macular regions.

## Introduction

In the last decade, technological advances in high-resolution imaging of the choroid using optical coherence tomography (OCT)^1,2^ have prompted a large number of studies to examine the morphology of the choroid in a range of ocular conditions^3–20^. Choroidal thickness varies substantially across the macula (the central 5 mm region)^21^, reducing from the fovea (the central 1 mm region) towards the outer macular regions (a 1–5 mm annulus including the parafovea and perifovea), and generally exhibiting a thicker profile superiorly and a thinner profile nasally and inferonasally^3–11^. The choroid also thins in myopia, more so in the foveal than the outer macular regions^4,8,9,11,18^. While these studies have substantially improved our understanding of the normal thickness of the choroid over the central macular zone (~17° area), the choroidal thickness profile beyond the macular region has not been investigated in detail.

Recent clinical studies suggest that the contribution of the choroid to the pathophysiology of a range of ocular diseases such as age-related macular degeneration^22^, central serous chorioretinopathy^23^, and glaucoma^24^ are not restricted to the macular region. However, in the absence of normative thickness data for the peripheral choroid, it is difficult to attribute changes in peripheral choroidal thickness solely to disease processes. Park *et al*.^25^ explored the variations in choroidal thickness across a ~40° area, centred on the optic nerve head, in a sample of healthy adults and found a thickening of the choroid in the superior region. However, measurements of choroidal thickness were limited to discrete locations across widely separated (~3 mm) OCT line scans and the regional distribution of choroidal thickness could not be examined comprehensively. In a pilot study, Mohler *et al*.^22^ examined the wide-field choroidal thickness of nine subjects with and without chorioretinal diseases, employing a laboratory prototype ultra-high speed swept-source OCT instrument, and reported substantial variation in peripheral choroidal thickness across a 60° field in both groups based on qualitative examination of the thickness maps.

Given the limited number of studies examining peripheral choroidal thickness in healthy subjects, and the paucity of knowledge regarding the normal distribution of peripheral choroidal thickness and its association with refractive error, the current study aimed to examine the wide-field choroidal thickness in healthy myopic and emmetropic young adults using wide-field enhanced depth imaging (EDI) OCT.

## Methods

### Subjects

This prospective cross sectional study enrolled 27 healthy young adults with a mean age of 27 ± 5 years recruited from the staff and students of the Queensland University of Technology (QUT), Brisbane, Australia. The mean refractive error of the participants was −0.94 ± 1.65 D (median: −0.50 D, range: −5.50 to +0.75 D; based on non-cycloplegic subjective refraction), with 14 emmetropic subjects (spherical equivalent refraction between −0.50 and +0.75 D inclusive; mean ± SD: +0.25 ± 0.42 D, median: +0.25 D, range: −0.50 to +0.75 D) and 13 myopic subjects (spherical equivalent refraction between −0.75 and −6.00 D inclusive^18,26,27^; mean ± SD: −2.23 ± 1.51 D, median: −0.75 D, range: −0.75 to −5.50 D) who were matched for age (mean age of emmetropes 28 ± 6 years, and myopes 25 ± 4 years, p = 0.3) and sex (50% male emmetropes and 46% male myopes, p = 0.8). All participants were non-smokers with no history of ocular or systemic pathology and were not using any medications. The participants had normal levels of stereoacuity (<60″ tested at 40 cm using the TNO test) and healthy corneae with no contraindication to contact lens wear based on slit lamp biomicroscopy. None of the myopic participants were under any myopia control treatment (e.g. atropine, orthokeratology, or multifocal spectacle or contact lenses). All subjects exhibited less than 0.75 D of hyperopia, 6.00 D of myopia, 1.00 D of astigmatism, and 1.00 D of anisometropia and all exhibited visual acuity of 0.00 logMAR or better. The majority of participants were Caucasian (n = 13), and the remaining participants had Indian (n = 6), East Asian (n = 5), and Middle Eastern (n = 3) ethnic backgrounds. Written informed consent was obtained from all participants and the study procedures were approved by the QUT Human Research Ethics Committee and adhered to the tenets of the Declaration of Helsinki.

### Data collection procedures

The right eye of each participant was examined using an EDI OCT (Spectralis HRA + OCT, Heidelberg Engineering Co, Heidelberg, Germany) with a wide-field lens module which provides repeatable measures of choroidal thickness in the macular and extra-macular regions^28,29^. A high-resolution (1536 × 496 pixels per B-scan) volumetric (including 37 horizontal line scans each being an average of 30 B-scans)^29^ scanning protocol was used to scan a 55° wide × 45° high area of the choroid centred on the fovea. During OCT imaging, both eyes were open and optimally corrected using a daily disposable soft contact lens (Proclear Daily, CooperVision Inc., Trumbull, CT, USA; power range: +0.75 to −5.25 D) to minimise the influence of image defocus upon choroidal thickness^30–33^. The presence of the contact lens did not significantly affect OCT image quality or measures of choroidal thickness; in agreement with previous studies examining the effect of contact lens wear upon retinal nerve fibre layer thickness^34^ and macular retinal thickness^35^. The right eye (the eye being scanned) viewed the instrument’s internal fixation light positioned at optical infinity, and the fellow eye fixated the centre of a high-contrast Maltese cross at a 5 metre distance under a dichoptic viewing condition.

Optical biometry of the right eye was performed (with the correcting contact lens in place) using the Lenstar LS 900 optical biometer (Haag-Streit AG, Koeniz, Switzerland) to obtain measures of the ocular dimensions along the visual axis which were used to rescale the OCT images to account for the effect of ocular magnification (described below). The right eye contact lens was then removed and optical biometry was repeated on the bare right eye to acquire measures of the axial length defined as the distance from the anterior cornea to the retinal pigment epithelium. Five measures were collected (with and without the contact lens) and the average ocular biometry measurements were used for further analysis.

Diurnal variations in choroidal thickness^36,37^ and axial length^38^ were accounted for by collecting data at the same time of the day at least 2 hours after waking (mean ± SD, 10:51 AM ± 1:20 hours). The potential impact of near tasks^39,40^, physical activity^41^, and ambient lighting^42^ on measures of choroidal thickness and axial length was minimised by providing a 20-minute wash-out period prior to image acquisition during which the subjects watched a movie from a 5 metre distance in low photopic ambient light levels (10 lux) which were maintained throughout all imaging procedures. A larger pupil diameter associated with low photopic ambient lighting also facilitated the wide-field imaging of the choroid.

### Regional measurements of choroidal thickness

The influence of image magnification associated with ocular refraction and axial dimensions of the eye upon regional measures of choroidal thickness was minimised using a method described previously to adjust the transverse scale of the OCT scans to account for ocular magnification^18^. Given the presence of the corrective spherical contact lens during OCT imaging, measures of non-cycloplegic objective refraction and optical biometry were acquired from the right eye using the Shin-Nippon NVision–K5001 open-field autorefractometer (Shin Nippon, Tokyo, Japan) and Lenstar LS 900 respectively while the corrective contact lens was worn. Regional measurements of choroidal thickness were then extracted from these magnification adjusted 55° wide-field B-scans which varied from 15.2 to 18.3 mm in width across subjects.

**Figure 1:**
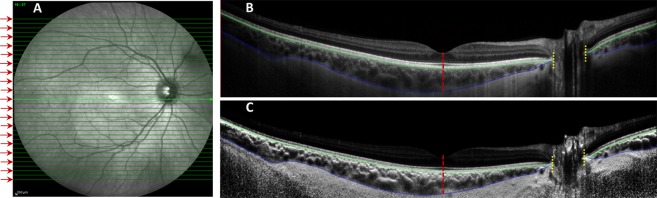
Illustration of the volumetric scanning protocol, which consisted of 37 high-resolution horizontal EDI B-scans (green lines) centred on the fovea for wide-field OCT imaging of the choroid across a 55° wide × 45° high region (**A**), with the thickness of the choroid analysed across every second line scan (red arrows). A representative trans-foveal B-scan is shown in (**B**) where an experienced masked observer marked the position of the fovea (red dashed line) and the optic nerve head boundaries (yellow dashed line) and segmented the anterior (green line) and posterior (blue line) boundaries of the choroid using a semi-automatic procedure. If the posterior choroidal boundary was difficult to delineate (**B**), image contrast enhancement was used to improve the visualisation of the chorioscleral junction (**C**).

The anterior and posterior boundaries of the choroid‚ defined as the outer border of the hyper-reflective line corresponding to the RPE/Bruch’s membrane complex‚ and the inner border of the hyper-reflective line corresponding to the chorioscleral junction, respectively, were delineated in every second volumetric B-scan (i.e. 19 B-scans from the 37-line volumetric scan) by an experienced masked observer using customised software (Fig. 1)^43,44^. If the posterior boundary of the choroid was difficult to delineate, image contrast enhancement was used to improve the visualisation of the chorioscleral junction (Fig. 1C)^45^. The position of the fovea, defined as the deepest point of the foveal pit, was marked manually on the trans-foveal B-scan, and the optic nerve head boundaries, characterised as the termination of Bruch’s membrane, were manually marked on the B-scans traversing the optic nerve to exclude this area from further analyses due to the absence of choroidal tissue.

Conventional thickness metrics may not provide accurate measures of wide-field choroidal thickness due to image tilt and curvature of the peripheral regions of wide-field OCT B-scans associated with retinal curvature and irregularities of the posterior choroidal boundary typically observed in the peripapillary region and areas adjacent to the insertion of the ciliary arteries and inferior oblique muscle^42^. Therefore, the Laplace method, commonly used in thickness measurements of convoluted brain tissue^46^ (and recently employed in measurements of wide-field choroidal thickness^29,42^, retinal thickness^42^, and contact lens thickness^47^) was employed to accurately estimate the regional thickness of the choroid^42^. This approach involves mathematical modelling of the thickness as a series of nested sublayers and calculates the thickness along a series of sequential lines perpendicular to each sublayer that connect the anterior and posterior boundaries of the choroid^46^; resulting in accurate estimates of choroidal thickness in areas with a non-uniform anterior/posterior border^29,42^. Wide-field choroidal thickness maps centred on the fovea were then generated for each participant using a bilinear interpolation algorithm.

**Figure 2:**
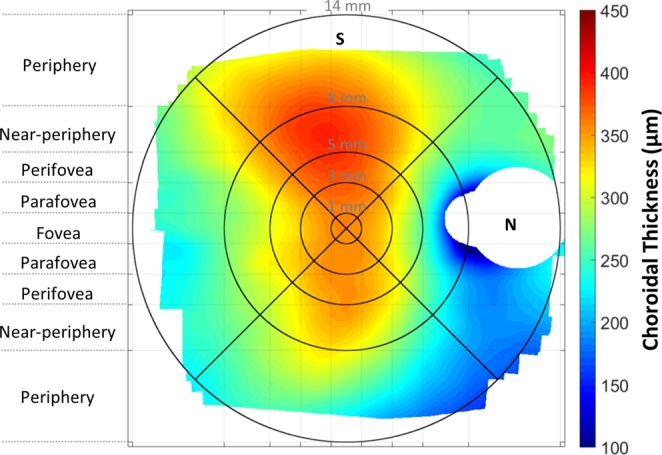
Group mean wide-field choroidal thickness map of all healthy young adults (n = 27). The white blank regions represent the areas with no common data across the optic nerve head, the nasal near-peripheral (12% missing data within this area) and peripheral regions (33%) and also the extreme superior (27%), temporal (18%), and inferior (17%) peripheral regions. S, Superior choroid; N, Nasal choroid.

Given that the anatomical variation in the relative position of the fovea and optic nerve head^48^ results in a potential source of individual variability in regional measures of choroidal thickness, the variability in the disc-fovea angle (defined as the angle between the horizontal trans-foveal line and the line connecting the centre of the optic disc to the fovea) was accounted for by rotating the en-face retinal image obtained during the OCT imaging (and the corresponding choroidal thickness map) around the fovea to reduce this angle to zero for all subjects^49^. These individual, anatomically-normalised, and magnification corrected wide-field choroidal thickness maps were then averaged over common choroidal locations across all subjects to provide a group mean wide-field choroidal thickness map and mean wide-field choroidal thickness maps for the emmetropic and myopic refractive error groups. The average thickness of the choroid was estimated across concentric annuli in the macular and extra-macular regions within the nasal, superior, temporal and inferior quadrants using the average choroidal thickness maps (Fig. 2). The macular region included the foveal, parafoveal, and perifoveal eccentricities (1 mm, 3 mm, and 5 mm in diameter centred on the fovea respectively) and the extra-macular region included the near-peripheral, and peripheral eccentricities (8 mm and 14 mm in diameter centred on the fovea respectively).

### Data analysis

Regional variations in choroidal thickness and their association with myopia was examined using a repeated measures analysis of variance (ANOVA) including two within subject factors of choroidal eccentricity (5 levels) and quadrant (4 levels) and a between subject factor of refractive error group (2 levels). To visualise and quantify the statistical significance of local changes in choroidal thickness associated with refractive error, measures of choroidal thickness at individual choroidal locations across the wide-field map were compared between emmetropes and myopes using a Student t-test. A wide-field p-value map was also generated to highlight areas of significant difference associated with myopia. The association between choroidal thickness at each eccentricity with both axial length and spherical equivalent refractive error was examined using Pearson’s correlation analysis.

## Results

The regional variations in measures of wide-field choroidal thickness and the mean wide-field topographic choroidal thickness map in this cohort of healthy young adults are presented in Table 1 and Fig. 2 respectively. The mean wide-field choroidal thickness (averaged across the entire 55° field) was 323 ± 70 μm and varied significantly with eccentricity and quadrant (both p < 0.001). While measures of foveal (central 1 mm region, 355 ± 90 μm) and parafoveal (1–3 mm annulus, 350 ± 86 μm) choroidal thickness did not differ (p = 0.64), the choroid progressively thinned beyond the parafovea, across the perifoveal (3–5 mm annulus, 336 ± 79 μm) and near-peripheral (5–8 mm annulus, 306 ± 65 μm) eccentricities, reaching a minimum in the periphery (8–14 mm annulus, 264 ± 44 μm) (p < 0.01 for all pairwise comparisons). The nasal (290 ± 79 μm) and superior choroid (355 ± 76 μm) exhibited the thinnest and thickest thickness profiles respectively (p < 0.01 for all pairwise comparisons), with no significant difference in choroidal thickness observed between the temporal (320 ± 60 μm) and inferior (322 ± 73 μm) quadrants (p = 0.9).

**Table 1:** Overview of the regional variations in wide-field choroidal thickness in emmetropes and myopes.

	Mean ± SD choroidal thickness (μm)		
	All subjects (n = 27)	Emmetropes (n = 14)	Myopes (n = 13)
Wide-field*	323 ± 70	351 ± 72	293 ± 80^†^
**Eccentricity**			
Fovea	355 ± 90	394 ± 70	316 ± 94^†^
Parafovea	350 ± 86	386 ± 68	312 ± 89^†^
Perifovea	336 ± 79	368 ± 61	303 ± 84^†^
Near-periphery	306 ± 65	329 ± 51	282 ± 71
Periphery	264 ± 44	277 ± 37	251 ± 48
**Quadrant**			
Superior	355 ± 76	385 ± 60	325 ± 81†
Temporal	320 ± 60	342 ± 47	299 ± 66†
Inferior	322 ± 73	356 ± 55	289 ± 75†
Nasal	290 ± 79	321 ± 66	259 ± 82†

*Choroidal thickness averaged across the 55° wide-field region centred on the fovea; ^†^Significantly thinner choroid in the myopic compared to the emmetropic group (p < 0.05).

**Figure 3:**
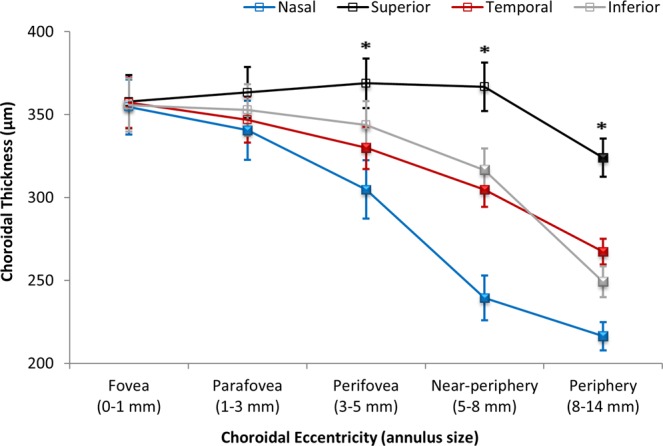
Mean choroidal thickness (μm) measured across the 55° area across different quadrants as a function of choroidal eccentricity. Error bars represent the standard error of the mean. Asterisks denote a statistically significant difference (p < 0.05) between the superior and the remaining quadrants. Filled symbols denote a statistically significant difference (p < 0.05) in a given choroidal eccentricity compared to the adjacent eccentricity to the left.

The magnitude of choroidal thinning into the periphery varied significantly with fundus quadrant (p < 0.001) (Fig. 3). Significant choroidal thinning was observed across all eccentricities in the nasal quadrant compared to the fovea (central 1 mm), with this nasal choroidal thinning being most pronounced in the near-peripheral (5–8 mm annulus, 239 ± 73 μm) and peripheral regions (8–14 mm annulus, 219 ± 48 μm). However, choroidal thickness in the superior quadrant did not vary significantly across the central macular (central 5 mm, fovea to parafovea and perifovea) and near-peripheral regions (ranging from 356 ± 90 to 365 ± 79 μm in the fovea to the near-periphery) and only exhibited significant thinning in the outermost peripheral eccentricity (323 ± 62 μm).

**Figure 4:**
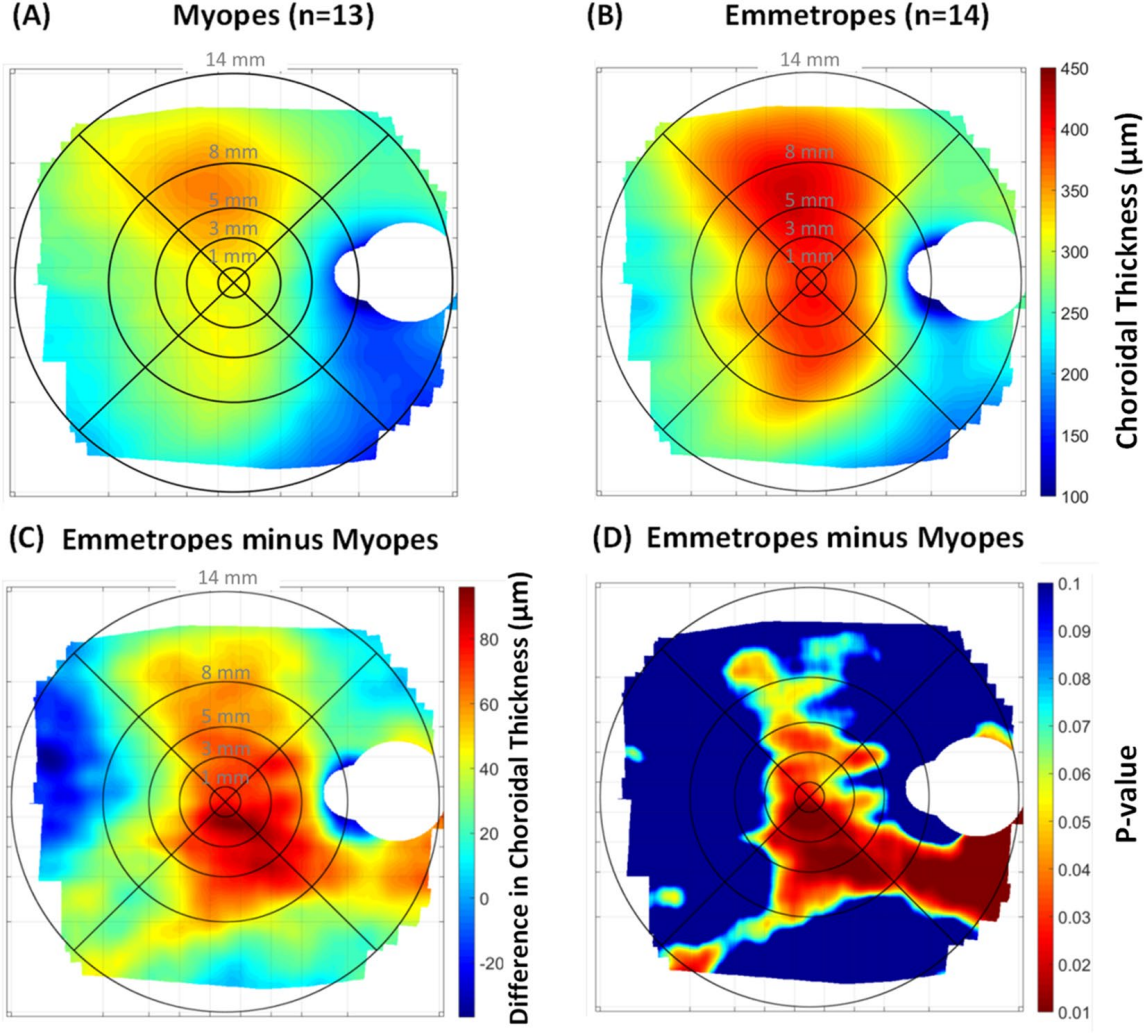
Mean wide-field choroidal thickness in myopic (n = 13) (**A**) and emmetropic (n = 14) (**B**) healthy young adults. The mean difference in wide-field choroidal thickness between emmetropes and myopes is shown in (**C**), where red indicates a thicker choroid in emmetropes compared to myopes, and blue indicates a thinner choroid in emmetropes compared to myopes. The regional variations in statistical significance of the observed variation in choroidal thickness associated with refractive error is shown in (**D**) where p-values represent the significance of point-wise Student t-test analyses performed across the fundus.

**Figure 5:**
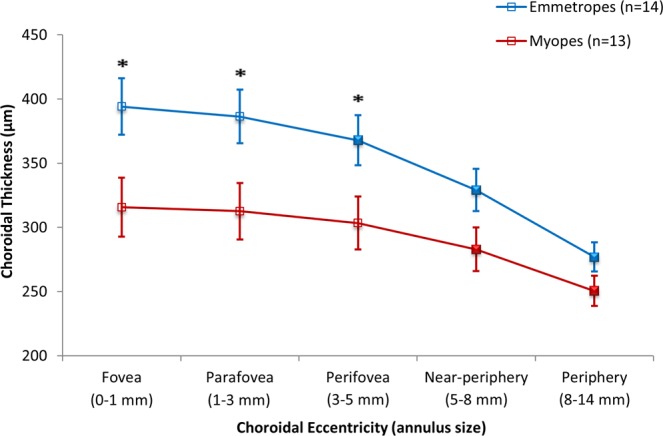
Mean choroidal thickness (μm) measured across different eccentricities over the central 55° area of the fundus in emmetropes (n = 14) and myopes (n = 13). Error bars represent the standard error of the mean. Asterisks and filled symbols denote a statistically significant difference (p < 0.05) between emmetropes and myopes, and between choroidal thickness at a given eccentricity compared to the adjacent eccentricity to the left in each refractive error group respectively.

Wide-field choroidal thickness also varied significantly with refractive error group (p = 0.03), with myopes (293 ± 80 μm) exhibiting a thinner choroid compared to emmetropes (351 ± 72 μm) when averaged across the 55° central area of the fundus (Table 1). While variations in choroidal thickness between quadrants were not influenced by refractive error group (p = 0.18), eccentricity-dependent variations in choroidal thickness were observed between the myopes and emmetropes (p = 0.004) (Fig. 4). In emmetropes, a significant thinning of the choroid was observed beyond the parafovea (386 ± 68 μm) into the periphery (277 ± 37 μm) across a 3–14 mm annulus; while myopes exhibited a significant reduction in choroidal thickness at the two outermost near-peripheral (282 ± 71 μm) and peripheral (251 ± 48 μm) regions across a 5–14 mm annulus. The significant choroidal thinning associated with myopia was confined to the macular region (central 5 mm region), with differences in choroidal thickness between emmetropes and myopes in the near-peripheral (329 ± 51 Vs. 282 ± 71 μm, p = 0.06) and peripheral (277 ± 37 Vs. 251 ± 48 μm, p = 0.13) regions not reaching statistical significance (Fig. 5).

As expected, on average, myopes (24.8 ± 1.4 mm) exhibited a significantly longer axial length than emmetropes (23.6 ± 0.8 mm) (p = 0.01). A statistically significant negative association was observed between axial length and choroidal thickness within the macular region (fovea, parafovea and perifovea all p ≤ 0.05), however, this association was not statistically significant for measures of extra-macular choroidal thickness (p > 0.05). Likewise, a positive association was observed between the central spherical equivalent refractive error and choroidal thickness within the macular region (all p < 0.05), but not for measures of extra-macular choroidal thickness (p > 0.05).

## Discussion

Numerous studies have characterised the normal thickness of the choroid in healthy young adults using EDI OCT^3–11,21^. However, the scanning and measurement protocols used in these studies have limited the quantification of choroidal thickness to the macular region (the central ~17°). In this study, we have extended the understanding of the normal *in-vivo* structure of the choroid beyond the macular region in healthy young adults by examining the regional variations in choroidal thickness over a much larger area of the fundus (~55°) than the majority of previous studies. Given the limited understanding of the impact of myopia on the morphology of the choroid beyond the macular region, we have further examined the regional changes in wide-field choroidal thickness associated with refractive error in this sample of healthy emmetropic and myopic young adults.

A number of studies have described the spatial variations in choroidal thickness within the macular area^3–11^, with the thickness of the choroid increasing significantly in the superior region and thinning markedly in the nasal quadrant. Our study supports these previous findings, with the superior (355 ± 76 μm) and nasal (290 ± 79 μm) choroid exhibiting the thickest and thinnest profiles respectively across a 55° area. We found significant eccentricity-dependent variations in choroidal thickness, with progressive choroidal thinning observed beyond the parafovea (350 ± 86 μm) into the periphery (264 ± 44 μm). Choroidal thinning of 2–16% from the fovea towards the perifovea has been reported in previous studies of healthy adults^3–11^. Consistent with these findings, a 5% reduction in choroidal thickness was observed from the fovea (355 ± 90 μm) towards the perifovea (336 ± 79 μm) in our study. We have further demonstrated that a more pronounced thinning of the choroid occurs in the near-peripheral (306 ± 65 μm) and peripheral (264 ± 44 μm) regions, with a 26% reduction in choroidal thickness observed in the periphery compared to the central foveal region.

Tanabe *et al*.^7^ found that the peripheral thinning of the choroid was more pronounced in the nasal and inferior aspects of the posterior pole, and Ouyang *et al*.^5^ observed a thickening of the choroid in the superior outer macular regions (rather than a thinning), suggesting an asymmetric variation in macular choroidal thickness that interacts with both choroidal eccentricity and quadrant. Our examination of choroidal thickness across a wider retinal area also suggests that the peripheral thinning of the choroid varies substantially between quadrants. In the superior quadrant, the choroid exhibited a slight thickening of approximately 2% from the fovea towards the near-periphery, but a 10% thinning was observed at the outermost peripheral region relative to the fovea. In contrast, the choroid progressively thinned from the fovea towards the periphery across all other quadrants, ranging from 25% to 30% in the temporal and inferior quadrants and reaching a maximum of 40% in the nasal quadrant.

Given that our study participants were healthy young adults without chorioretinal pathology, the significant peripheral thinning of the choroid observed in our study reflects the normal anatomical structure of the choroid across the central 55°. This peripheral thinning of the choroid generally follows the spatial distribution of the density of vascular layers of the choroid, particularly the outer larger vascular layer^22,50,51^. Examination of the mean wide-field choroidal thickness map (Fig. 2) reveals a prominent thickening of the choroid in the superior quadrant and a distinct thinning in the nasal quadrant that extends inferiorly towards the periphery. Given that the spatial location of the choroidal veins in the posterior pole correlate with regional changes in macular choroidal thickness^52^, the spatial distribution of these larger lumen veins in the choroid may contribute to the wide-field distribution of choroidal thickness observed in our study. A previous study of the choroid in healthy human eyes using indocyanine green angiography found that preferential superior choroidal venous drainage was the most common feature of the venous vascular structure of the choroid^53^. This most likely explains the greater thickness of the superior choroid (and least peripheral thinning in this region) observed in our study. Further, the peripapillary and inferior-nasal thinning of the wide-field choroid found in our study is consistent with previous studies examining choroidal thickness in this region^7,54^ and can be explained by the anatomical location of the watershed zones of the choroid vasculature, typically passing vertically through the optic nerve^55^. The inferior-nasal location of the embryologic closure of the optic fissure may also contribute to the observed nasal and inferior-nasal choroidal thinning^7,13^.

Wide-field choroidal thickness averaged across the entire 55° region was 16% thinner in myopic young adults (293 ± 80 μm) compared to emmetropes (351 ± 72 μm), consistent with previous studies of macular choroidal thickness in adults^3,4,6,8,9,11–13^ and children^14–18^. This finding provides further evidence regarding the significant contribution of myopia to the variations in the thickness of the choroid across a wider area of the fundus. While previous studies have reported an association between choroidal thickness and axial length or refractive error across the macula^3–6^, our results further revealed that myopia and increased axial length were associated with significant thinning of the choroid primarily in the macular compared to the extra-macular regions (Fig. 5). While the choroid was 19% thinner in myopes compared to emmetropes in the macular region, it was only 10% thinner in the periphery. Previous studies of the influence of adult myopia on macular choroidal thickness have found between 18% to 23% thinning of the macular choroid associated with myopia, with the foveal choroidal thickness typically exhibiting more thinning with myopia (20% to 26%) compared to the perifoveal region (16% to 20%)^8,9,11^.

Read *et al*.^18^ and Vincent *et al*.^56^ calculated that the thinning of the choroid associated with myopia is only partially explained by a passive mechanism resulting from a myopic expansion of the eye in children and adults respectively. Likewise, established myopia in our sample of healthy young adults was associated with a 20% thinning of the foveal choroid which substantially exceeded the magnitude of thinning predicted from a simple mechanical stretching of the eye (given the 5% longer axial length in the myopic subjects compared to the emmetropic group). This suggests that additional active mechanisms may contribute to alterations in choroidal thickness associated with myopia and is consistent with evidence of active modulation of the choroidal thickness in the development of experimental myopia observed in animals^57^, short-term defocus studies in humans^29–32,58^ and also the potential role of the choroid in the regulation of eye growth in children^59^. The spatial anatomical arrangements of the vascular^51^ and contractile non-vascular cells^60^ in the choroid along with the distribution of the choroidal intrinsic ganglion cells^61^, that are generally found in greater densities in more central regions of the choroid, may imply that the central choroid has a greater contribution to active choroidal changes associated with myopia development compared to the periphery. Collectively, these results suggest that the reduction in choroidal thickness associated with myopia is largely confined to the macular region and that active mechanisms in the eye may underlie the structural changes in choroidal thickness associated with myopia.

In this study, the thinning of the choroid associated with myopia was greater inferiorly and nasally than superiorly and temporally (Fig. 4C,D). Previous studies have also reported an increase in choroidal thickness in the temporal region and a reduction in choroidal thickness in the nasal region in both moderate^62^ and highly myopic adults^63^. While the exact mechanism for this asymmetric profile in choroidal thickness is not understood, it has been speculated that mechanical forces associated with myopic axial elongation exerted at the temporal aspect of the optic nerve (nasal to the fovea) may ultimately cause a thinning of the nasal choroid along with a displacement of the thicker foveal choroid temporally^49,62^. Likewise, it may be reasonable to hypothesise that the greater thinning of the inferior choroid in myopes than emmetropes may be attributed to a vertical asymmetry in mechanical forces associated with myopic axial elongation of the eye.

The effect of myopia on the spatial distribution of macular choroidal thickness has been studied in two paediatric populations with a mean age of 10^14^ and 13 years^18^, with the foveal choroid exhibiting a 10–16% reduction in thickness associated with myopia, which is less than the magnitude of foveal choroidal thinning observed in this study (20%) and other studies of young adults (20–26%)^8,9,11^. Despite a fairly similar magnitude of myopia across these studies of children and adults, a greater thinning of the foveal choroid is observed in older groups. Previous large scale cross sectional studies of sub-foveal choroidal thickness have also reported a greater thinning of the choroid with each dioptre of myopia in adults (average 15.7 μm per dioptre)^64^ compared to children (average 9.5 μm per dioptre)^16^. While the reason for the greater reduction in choroidal thickness of myopic adults compared to children with a similar magnitude of myopia is unclear, age dependent changes in the choroidal contribution to the development of refractive errors may be a relevant factor. If the thinning of the choroid actively contributes to, or acts as a biomarker for processes underlying the development of myopia, a greater choroidal thinning in young adults compared to children, in the presence of similar magnitude of myopia in both age groups, implies that the strength of the choroidal contribution to myopia development reduces with age. Vision dependent mechanisms regulating refractive error development in animals appear to be less potent with increasing age^65^, however, age-dependent changes in the contribution of the choroid to visually guided mechanisms of eye growth remain largely unexplored.

Similar to studies of young adults, choroidal thinning in myopic children (mean age 13 years) was greater at the fovea compared to the perifovea^18^. However, in a younger population of myopic children (mean age 10 years) choroidal thinning was consistent across the macular region^14^. Although differences in imaging protocols and population characteristics other than age may contribute to the pattern of change in choroidal thickness associated with myopia observed in the studies of children and adults, it appears that the eccentricity-dependent thinning of the choroid associated with myopia may occur during the early teenage years and persist throughout adulthood. Currently, there is a lack of knowledge regarding the wide-field choroidal thickness at different stages of ocular development and future longitudinal studies are needed to determine the spatial characteristics of the contribution of the choroid to myopia development across a wide area of the fundus in paediatric populations.

This study was conducted on a relatively small cohort of emmetropic and low to moderate myopic young adults and was limited by its cross sectional design. Therefore, the results may not be able to be generalised to larger populations of varying ages, ethnicities and refractive errors (e.g. hyperopia, astigmatism, and high myopia). Although measurements were collected at the same time of the day to limit the effect of diurnal changes, measurements of intraocular pressure and hemodynamic variables including the mean arterial pressure and ocular perfusion pressure, which have been suggested to correlate with measurements of choroidal thickness in the fovea, were not captured^66^. The missing data reported in the mean wide-field choroidal thickness maps in the optic nerve head area and extreme regions of the peripheral eccentricity may have also limited the results. However, analysis performed on data using a smaller annulus for the peripheral eccentricity (4–6 mm compared to 4–7 mm annulus) to reduce the missing data in the nasal, superior, temporal, and inferior peripheral quadrants to 27%, 1%, 0.5%, and 0% (compared to 33%, 27%, 18%, and 17% originally) revealed no change in the statistically significant main effects and interactions compared to the original analysis. Therefore, the missing data reported in the peripheral regions has minimal influence upon the conclusions of this study. Further, any causal relationship between the observed changes in wide-field choroidal thickness and myopia could not be assessed. Future longitudinal experiments examining alterations in macular and peripheral choroidal thickness during myopia development and progression in younger cohorts of children should provide a better understanding of the role of the choroid across the wide-field area in myopic eye growth.

In summary, the choroid exhibited significant peripheral thinning that varied with choroidal quadrant; a 40% reduction in choroidal thickness was observed nasally from the fovea towards the periphery, compared to 10% in the superior quadrant. Variations in choroidal thickness were also associated with refractive error. Established myopia in young adults was associated with a significantly thinner choroid within the central macular region, and differences in choroidal thickness associated with myopia diminished in the periphery. This regional variation could reflect greater myopiagenic activity in the central retina compared to the periphery.

## Data Availability

The datasets generated and analysed during this study are available from the corresponding author on reasonable request.
